# CHI3L2 Is a Novel Prognostic Biomarker and Correlated With Immune Infiltrates in Gliomas

**DOI:** 10.3389/fonc.2021.611038

**Published:** 2021-04-15

**Authors:** Liling Liu, Yuanzhong Yang, Hao Duan, Jiahua He, Lu Sun, Wanming Hu, Jing Zeng

**Affiliations:** ^1^ Department of Pathology, Sun Yat-Sen University Cancer Center, Guangzhou, China; ^2^ State Key Laboratory of Oncology in Southern China, Sun Yat-Sen University Cancer Center, Guangzhou, China; ^3^ Department of Neurosurgery, Sun Yat-Sen University Cancer Center, Guangzhou, China

**Keywords:** CHI3L2, gliomas, CGGA, TCGA, prognosis, immune infiltrates

## Abstract

CHI3L2 (Chitinase-3-Like Protein 2) is a member of chitinase-like proteins (CLPs), which belong to the glycoside hydrolase 18 family. Its homologous gene, CHI3L1, has been extensively studied in various tumors and has been shown to be related to immune infiltration in breast cancer and glioblastoma. High CHI3L2 expression was reported to be associated with poor prognosis in breast cancer and renal cell carcinoma. However, the prognostic significance of CHI3L2 in glioma and its correlation between immune infiltration remains unclear. In this study, we examined 288 glioma samples by immunohistochemistry to find that CHI3L2 is expressed in tumor cells and macrophages in glioma tissues and highly expressed in glioblastoma and IDH wild-type gliomas. Relationships between CHI3L2 expression and clinical features (grade, age, Ki67 index, P53, PHH3 (mitotic figures), ATRX, TERTp, MGMTp, IDH, and 1p/19q co-deleted status) were evaluated. Kaplan-Meier survival was conducted to show high CHI3L2 expression in tumor cells (TC) and macrophage cells (MC) indicated poor prognosis in diffusely infiltrating glioma (DIG), lower-grade glioma (LGG), and IDH wild-type gliomas (IDH-wt). The overall survival time was higher in patients with dual-low CHI3L2 expression in TC and MC compared to those in patients with non-dual CHI3L2 expression and dual high expression in DIG and IDH wild-type gliomas. By univariate and multivariate analysis, we found that high CHI3L2 expression in tumor cells was an independent unfavorable prognostic factor in glioma patients. Moreover, we used two datasets (TCGA and CGGA) to verify the results of our study and explore the potential functional role of CHI3L2 by GO and KEGG analyses in gliomas. TIMER platform analysis indicated CHI3L2 expression was closely related to diverse marker genes of tumor immune infiltrating cells, including monocytes, TAMs, M1 macrophages, M2 macrophages, TGFβ1+ Treg and T cell exhaustion in GBM and LGG. Western Blot validated CHI3L2 is expressed in glioma cells and microglia cells. The results of flow cytometry showed that CHI3L2 induces the apoptosis of CD8+ T cells. In conclusion, these results demonstrate CHI3L2 is related to poor prognosis and immune infiltrates in gliomas, suggesting it may serve as a promising prognostic biomarker and represent a new target for glioma patients.

## Introduction

Gliomas comprise the bulk of primary brain tumors in adults (1). Diffuse glioma is histopathologically classified into grade II-IV according to morphological criteria, including mitotic count, nuclear atypia, microvascular proliferation, and necrosis. Glioblastoma multiform (GBM) is categorized as one of the most malignant subtypes (2, 3). The 2016 World Health Organization (WHO) classification of adult diffuse glioma combines tumor histological morphology and molecular features, including the isocitrate dehydrogenase (IDH) mutation and the chromosomal arms 1p and 19q complete deletion (1p/19q co-deletion) (4). Even combining maximal surgical resection and radiotherapy with adjuvant temozolomide, tumor recurrence is inevitable and the prognosis of gliomas remains very poor (5). Consequently, it is an urgent demand to discover the potential molecular characteristics of gliomas and look for more effective treatment strategies.

CHI3L2 (Chitinase-3-Like Protein 2), also known as YKL39, is a kind of secretory protein. It is a member of chitinase-like proteins (CLPs) which include CHI3L1, CHI3L2, SI-CLP, YM1 and YM2. CHI3L2 was originally isolated from the culture medium of primary human articular cartilage cells (6). It has two physiological activities, one is to induce autoimmune response (7), the other is to participate in tissue remodeling, both of which may lead to disease progression. Previous studies showed CHI3L2 mRNA is significantly up-regulated in osteoarthritis, Alzheimer's disease, multiple sclerosis, and amyotrophic lateral sclerosis patients (8–11). CHI3L2 was secreted by microglia/astrocytes and could increase monocyte/macrophage infiltration, angiogenesis, and neuronal death in amyotrophic lateral sclerosis (11). It is not yet clear what type of cells CHI3L2 is expressed in gliomas. However, previous studies on CHI3L2 have shown that macrophages are a possible source of CHI3L2 in tumors (12–15). CHI3L2 has a high degree of sequence identity with CHI3L1, but no cross-reactivity has been observed (16–18). There have been many studies on the correlations between CHI3L1 and the progression of a number of cancers (19–22). In recent years, the relationship between tumor immune microenvironment and immunotherapy has received more and more attention. It was also reported CHI3L1 was related to immune infiltration in breast cancer and glioblastoma (23, 24). However, the data about the role of CHI3L2 in cancers and its association with immune infiltrates are fragmentary. Previous studies reported that CHI3L2 was overexpressed in tumor-associated macrophages and related to poor outcomes in breast cancer and renal cell carcinoma (13–15). Studies have been shown that CHI3L2 mRNA expression was increased in gliomas (18, 25, 26). However, the prognostic significance of CHI3L2 and its correlation with immune infiltrates in glioma remain unclear.

To systematically explore the CHI3L2 protein expression in diffusely infiltrating glioma, we first evaluated the CHI3L2 expression levels of 288 glioma tissues by immunohistochemistry (IHC) and analyzed the association between CHI3L2 levels and clinicopathological parameters. Moreover, we took advantage of CHI3L2 transcriptional data of gliomas in The Cancer Genome Atlas (TCGA) and the Chinese Glioma Genome Atlas (CGGA) datasets to validate our findings. Gene Ontology (GO) and Kyoto Encyclopedia of Genes and Genomes (KEGG) pathway analyses were used to explore the potential biological process and pathways of CHI3L2 in glioma. The Tumor IMmune Estimation Resource (TIMER) platform was used to explore the correlations between CHI3L2 and diverse marker genes of tumor immune infiltrates. Finally, we further verified the results through Western Blot and flow cytometry. In gliomas, this is the first comprehensive study to elaborate on the clinical significance of CHI3L2, its influence on prognosis and its correlation with immune infiltrates.

## Materials and Methods

### Samples

We enrolled 288 glioma patients (WHO grade II-IV) operated at the Sun Yat-sen University Cancer Center (Guangzhou, China) from January 2009 to January 2016. The median follow-up time was 54 months. Follow-up was last done in June 2019. The detailed clinical data are listed in **Table S1**. There were 167 males and 121 females. The median age of all patients at initial diagnosis was 43 years (range 7-78years). According to MRI imaging, 278 cases of glioma were located on the supratentorial and 10 cases of glioma were located infratentorial. 264 out of 288 patients received postoperative adjuvant treatment (radiotherapy or chemotherapy). The median overall survival time of all patients was 27 months (range 0-110 months). This cohort included 112 cases of astrocytoma, 45 cases of oligodendroglial gliomas and 131 cases of glioblastoma (WHO IV). All samples were ethically approved for use based on informed consent.

### Cell Culture

The human glioma cell lines and a human microglia cell line were purchased from the American Type Culture Collection resource center. The human glioma cells were maintained in Dulbecco's modified Eagle's medium (DMEM) supplemented with 10% heat-inactivated fetal bovine serum (FBS) and the human microglia cells were cultured in Minimum Essential Medium (MEM) with 10% FBS at 37 °C in a humidified incubator containing 5% CO2. Peripheral blood mononuclear cells (PBMC) were isolated by Ficoll-Hypaque density gradient centrifugation (Solarbio, Beijing, China). CD8+ T cell were separated by positive selection from PBMCs with CD8 magnetic beads and cultured in RPMI-1640 medium supplemented with 10% human serum, 5% L-glutamine-penicillin-streptomycin solution (Sigma-Aldrich, USA), CD3/CD28 antibody (Biolegend, USA) (25ul/ml) and IL-2 (100IU/ml) in 24-well plates. After culturing for 24 hours, add the corresponding concentration of human CHI3L2 protein (Sino Biological, Beijing, China) to T cells and culture for 72 hours at 37 °C in a humidified incubator containing 5% CO2.

### Immunohistochemistry (IHC), Molecular Genetics and Assessment Standard 

Immunohistochemistry was essentially performed as previously reported (27). These tissue specimens were incubated with CHI3L2 rabbit polyclonal antibody (#22164, SAB, Maryland, USA). Immunohistochemical evaluation was independently conducted by two pathologists blinded for patient characteristics and outcome, and CHI3L2 expression by tumor cells and macrophage cells was scored separately. The discrepancies were resolved by consensus under a microscope for multi-viewing. A semi-quantitative IHC scoring criterion was used to determine the CHI3L2 protein expression levels in tumor cells. The percentage of positive cells and staining intensity were assessed to improve accuracy. The percent positivity of staining cells range from 0 to 4: 0, none; 1, 1%-25%; 2, 26%-50%; 3, 51%-75%; 4, 76%-100%. The intensity of staining was graded from 0 to 3 (0, no staining; 1, weak; 2, moderate and 3, strong). Then, we obtained the final IHC score by multiplying the proportion score by the intensity score of staining. We chose 4.5, which was determined by the Youden index as an optimal cutoff point to separate low CHI3L2 expression (score of 0-4.5) from high CHI3L2 expression (score>4.5) in tumor cells. For the macrophage cells, we only count the number of CHI3L2 positive staining macrophages, regardless of the staining intensity. We designed 7.5 determined by Youden index as an optimal cutoff point to differentiate low expression (number≤7.5) from high expression (number>7.5) in macrophages.

The other antibody markers including PHH3, P53, Ki67, ATRX, CD163, CD4, CD8, and CD20 were also used by immunohistochemistry tests. We detected MGMT promoter methylated status, TERT promoters and IDH mutation status by Sanger sequencing. 1p and 19q deletion status was detected using fluorescent in situ hybridization (FISH). The detailed protocol and assessment standard were described as a previous study (28).

### Bioinformatic Analysis in Cancer Datasets

The CHI3L2 RNA-seq data were downloaded from http://www.cgga.org.cn/. We totally analyzed 601 TCGA RNA-seq cohort and 608 CGGA RNA-seq cohort of gliomas, ranging from WHO grade II to grade IV.

To identify the CHI3L2-related genes, the limma package of R software was used to screen out the differentially expressed genes (DEGs). The top 26 hub genes of the overlapping DEGs were built via the plug-in molecular complex detection and cytoHubba of Cytoscape. To explore the functions and pathways of CHI3L2-related genes, we performed GO and KEGG analyses on ClueGo and Metascape websites. The TIMER platform (https://cistrome.shinyapps.io/timer/) (29, 30) was performed to explore the association between CHI3L2 and marker sets of tumor immune infiltrates in GBM and LGG (31).

### Western Blot

Total protein was extracted from seven human glioma cell lines and one human microglia cell line (HMC3). 30 ug of protein was loaded onto 10% SDSPAGE and electrophoretically transferred to PVDF membranes. After blocking, the membranes were incubated with primary antibody against CHI3L2 (1:1000 dilutions, rabbit polyclonal anti-CHI3L2, #22164, SAB, Maryland, USA). The membranes were then incubated with horseradish peroxidase-linked anti-rabbit antibody (at a 1:3000 dilution, Santa Cruz Biotechnology, Inc., Santa Cruz, Calif., USA). B-Actin was served as a loading control.

### Flow Cytometry Analysis

Apoptosis was examined by flow cytometric analysis. Cells were collected, washed with PBS, and incubated with annexin V-FITC and PI (BD Biosciences, San Jose, CA, USA) for 15 minutes. Then cell apoptosis was analyzed by flow cytometry (FACS Calibur, Becton Dickinson, Sparks, MD) according to the manufacturer’s instruction. FlowJo (Treestar, USA) software was used for the analysis of flow cytometry data. The results are expressed as mean ± SD of three independent experiments.

### Statistical Analysis

GraphPad Prism 8 and SPSS 22 software were performed for statistical analyses. The measurement data are represented as mean ± SD. The Chi-square test was conducted to explore the correlations between CHI3L2 levels and clinicopathological features. Kaplan-Meier analysis was conducted for the overall survival of glioma patients with the log-rank test. The Cox proportional hazards regression model was used for univariate and multivariate analyses to evaluate the independence of CHI3L2 in predicting prognosis. The association between CHI3L2 and marker genes of immune infiltrating cells was assessed by Spearman's correlation coefficients. P < 0.05 was regarded as statistically significant.

## Results

### The Expression Levels of CHI3L2 in Glioma Samples and Its Correlation With Clinicopathological Parameters

**Figure 1:**
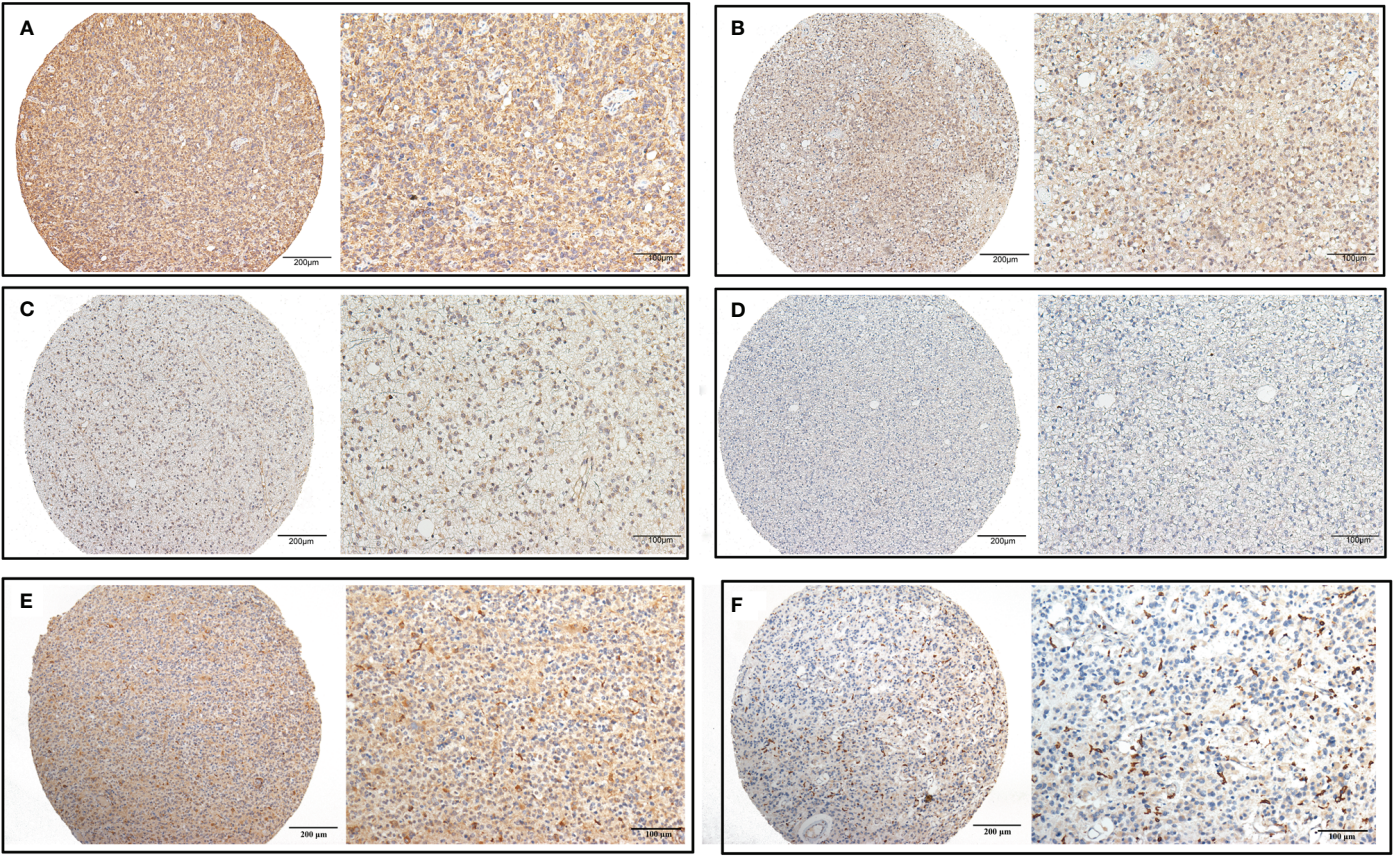
CHI3L2 protein expression was detected by IHC. Representative images of strong **(A)**, moderate **(B)**, weak **(C)**, and negative **(D)** staining of CHI3L2 are shown. Representative images of the high density of CHI3L2+ macrophages in strong **(E)** and weak **(F)** staining glioma tissues are shown.

**Figure 2:**
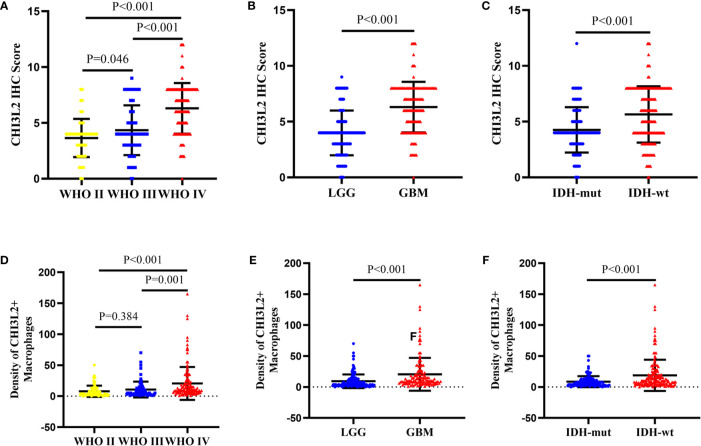
The protein expression levels of CHI3L2 in different subgroups of gliomas. **(A)** The IHC score in diffusely infiltrating glioma (WHO II-IV). **(B)** The IHC score in LGG and GBM. **(C)** The IHC score in diffusely infiltrating glioma with different IDH status. **(D)** The density of CHI3L2+ macrophages in diffusely infiltrating glioma (WHO II-IV). **(E)** The density of CHI3L2+ macrophages in LGG and GBM. **(F)** The density of CHI3L2+ macrophages in diffusely infiltrating glioma with different IDH status.

We detected CHI3L2 protein expression levels in histological sections from patients with different glioma grades by immunohistochemistry (IHC). Among the 288 glioma specimens inspected, we found CHI3L2 was mainly stained in tumor cells, as well as macrophage cells. **Figures 1A–D** showed different IHC staining intensity of CHI3L2 in glioma tissues. **Figures 1E, F** mainly showed CHI3L2+ macrophages in glioma tissues. The IHC score of CHI3L2 in tumor cells and density of CHI3L2+ macrophages in different glioma subgroups are shown in **Figure 2**. We found the expression levels of CHI3L2 in tumor cells were upregulated with increasing WHO grade of gliomas (**Figure 2A**), but there was no significant difference in CHI3L2+ macrophage density between WHO II and WHO III gliomas (**Figure 2D**). The CHI3L2 expression levels of GBM were significantly increased compared with LGG (WHO II-III) (P<0.001) in tumor cells (**Figure 2B**) and macrophages (**Figure 2E**). A significant increase of CHI3L2 expression levels was found in IDH-wildtype gliomas compared with IDH-mutant gliomas (P<0.001) in tumor cells (**Figure 2C**) and macrophages (**Figure 2F**). We further analyze the CHI3L2 IHC score and density of CHI3L2+ macrophages in diffusely infiltrating glioma of new molecular classification, including IDH mutant without 1p/19q codeleted gliomas, IDH mutant with 1p/19q codeleted gliomas, and IDH wild-type gliomas (**Figures S1A, B**). We found the expression of CHI3L2 is not related to the status of 1p/19q codeleted in IDH mutant gliomas.

Based on the expression levels of CHI3L2 in tumor cells and macrophage cells, we evaluated the association between CHI3L2 staining and clinicopathological factors, as listed in **Table 1**. In tumor cells, we found significant correlations between CHI3L2 expression and WHO grade (P<0.001), age (P=0.001), Ki67 (P<0.001), P53 (P=0.034), PHH3 (mitotic figures) (P<0.001), ATRX protein expression (P=0.026), IDH (P<0.001) and 1p/19q codeleted (P=0.002). In macrophage cells, CHI3L2 is strongly correlated with WHO grade (P<0.001), gender (P=0.008), age (P=0.006), Ki67 (P<0.001), PHH3 (mitotic figures) (P<0.001), and IDH status (P<0.001). However, CHI3L2 expression levels of glioma cells were not significantly related to gender, location, TERT promoter mutation status, and MGMT promoter methylated status. In macrophage cells, CHI3L2 expression has no correlation with location, P53, ATRX protein expression, MGMT promoter methylated status, TERT promoter mutation, and 1p/19q codeleted status.

**Table 1:** Correlation between CHI3L2 expression and clinicopathologic parameters in gliomas.

Parameters	CHI3L2 inTumor cells		P value	CHI3L2 inMacrophage cells		P value
	Low	High		Low	High	
Grade						
LGG	117	40	<0.001	96	61	<0.001
GBM	34	97		37	94	
Gender						
Male	81	86	0.117	66	101	0.008
Female	70	51		67	54	
Age						
<55	127	92	0.001	111	108	0.006
≥55	24	45		22	47	
Location						
Supratentorial	144	134	0.257	127	151	0.372
Infratentorial	7	3		6	4	
Ki67						
<10%	51	11	<0.001	42	20	<0.001
≥10%	100	126		91	135	
P53						
<10%	61	39	0.034	49	51	0.484
≥10%	90	98		84	104	
PHH3						
<5/10HPF	87	23	<0.001	67	43	<0.001
≥5/10HPF	64	114		66	112	
ATRX						
Negative	74	85	0.026	76	83	0.541
Positive	77	52		57	72	
TERTp						
Wild type	81	66	0.354	74	73	0.148
Mutant	70	71		59	82	
IDH						
Wild type	64	98	<0.001	59	103	<0.001
Mutant	87	39		74	52	
1p/19q Codeleted						
Yes	35	13	0.002	26	22	0.224
No	116	124		107	133	
MGMTp						
Methylated	73	69	0.732	66	76	0.920
Unmethylated	78	68		67	79	

P-value: Chi-square test.

### Impact of CHI3L2 Expression on the Prognosis of Gliomas

**Figure 3:**
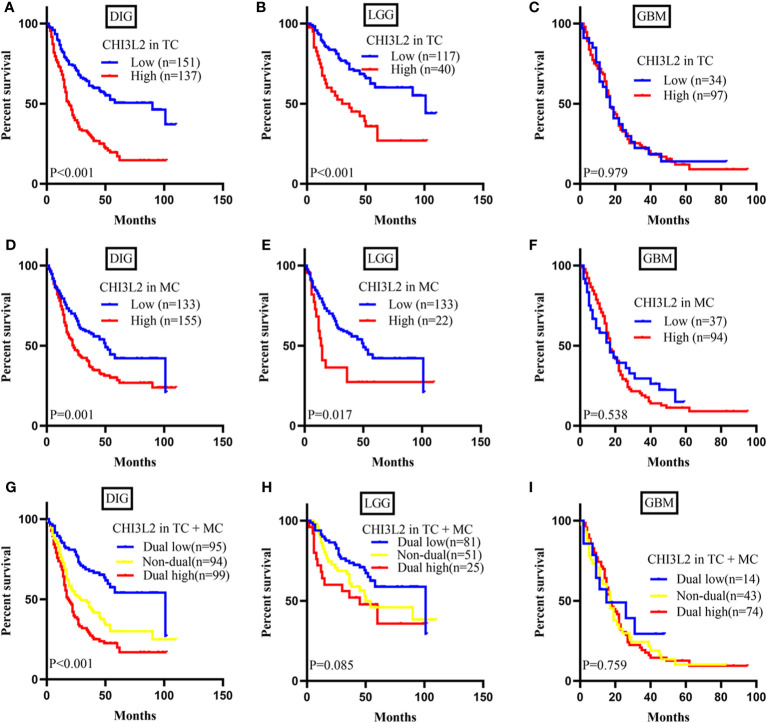
CHI3L2 protein expression affects overall survival (OS) in glioma patients. Kaplan-Meier curves showing a correction of CHI3L2 expression with OS in tumor cells **(A–C)**, macrophage cells **(D–F)**, and tumor cells + macrophages cells **(G–I)** of diffusely infiltrating glioma (DIG), LGG, and GBM.

To explore the prognostic significance of CHI3L2 in gliomas, we performed the Kaplan-Meier method and log-rank test. We found high CHI3L2 expression levels of tumor cells and macrophages significantly predicted worse overall survival in diffusely infiltrating glioma (DIG) (**Figures 3A, D**) and lower-grade glioma (LGG) patients (**Figures 3B, E**). However, there was no statistical significance difference in GBM in our cohort (**Figures 3C, F**). When considering the CHI3L2 expression of tumor cells and macrophages together, we found the overall survival time was higher in patients with dual-low CHI3L2 expression in TC and MC compared to those in patients with non-dual CHI3L2 expression and dual high expression in DIG (**Figure 3G**), but this difference is not statistically significant in LGG and GBM (**Figures 3H, I**). Similarly, we also analyze the effect of CHI3L2 on the prognosis in the new molecular classification of glioma. We found CHI3L2 expression in tumor cells is closely related to the prognosis of all new molecular classification of glioma, and high CHI3L2 expression in tumor cells, macrophages and TC + MC predicted poor outcome for IDH wild-type gliomas (**Figure S2**). Furthermore, we found regardless of whether patients with glioma have methylation of the MGMT promoter or have received adjuvant therapy, high CHI3L2 indicates a poor prognosis for glioma (**Figure S3**).

Additionally, to evaluate the independent risk factors for prognosis of glioma, we conducted the univariate analysis (**Table 2**) and multivariate analysis (**Table 3**). In univariate analysis, CHI3L2 expression in tumor cells, CHI3L2+ macrophage cells density, CHI3L2 expression in both tumor cells and macrophage cells (TC + MC), grade, age, location, adjuvant therapy, Ki67 index, PHH3 (mitotic figures), IDH, and 1p/19q codeleted status were shown to be prognostic variables for the prognosis of overall survival in glioma patients (**Table 2**). Then we included the prognostic variables in the univariate analysis (P<0.05) into the multivariate analysis. We found CHI3L2 expression in tumor cells, location, Ki67, IDH, 1p/19q codeleted were independent prognostic factors in gliomas (**Table 3**).

**Table 2:** Univariate analysis of prognostic variables of overall survival.

Variables	Univariate analysis		
	HR	95%CI	P
CHI3L2 expression in tumor cells (low vs. high)	2.564	1.890-3.479	**<0.001**
CHI3L2+ macrophage cells density (low vs. high)	1.650	1.217-2.238	**0.001**
CHI3L2 in TC + MC (dual low vs. dual high)	3.017	2.035-4.472	**<0.001**
CHI3L2 in TC + MC (dual low vs. non-dual)	2.094	1.396-3.140	**<0.001**
Grade (LGG vs. GBM)	3.642	2.663-4.983	**<0.001**
Gender (Male vs. Female)	0.747	0.551-1.014	0.061
Age (<55 vs. ≥55 years)	2.135	1.545-2.951	**<0.001**
Location (Supratentorial vs. Infratentorial)	2.374	1.165-4.837	**0.017**
Adjuvant therapy (no vs. yes)	3.036	1.421-6.485	**0.004**
Ki67 (<10% vs. ≥10%)	3.501	2.190-5.596	**<0.001**
P53 (<10% vs. ≥10%)	1.220	0.887-1.676	0.221
PHH3 (<5/10HPF vs. ≥5/10HPF)	2.756	1.963-3.868	**<0.001**
ATRX (negative vs. positive)	1.225	0.907-1.654	0.185
TERTp (wild-type vs. mutant)	1.136	0.845-1.527	0.399
MGMTp (methylated vs. unmethylated)	0.876	0.651-1.178	0.381
IDH (wild-type vs. mutant)	0.202	0.142-0.286	**<0.001**
1p/19q Codeleted (no vs. yes)	0.165	0.084-0.322	**<0.001**

**Table 3:** Multivariate analysis of prognostic variables of overall survival.

Variables	Multivariate analysis		
	HR	95%CI	P
CHI3L2 expression in tumor cells (low vs. high) CHI3L2+ macrophage cells density (low vs. high)	1.466 1.002	1.011-2.125 0.719-1.398	0.044 0.990
Grade (LGG vs. GBM)	1.017	0.604-1.715	0.949
Age (<55 vs. ≥55 years)	1.350	0.956-1.907	0.088
Location (Supratentorial vs. Infratentorial)	2.393	1.120-5.112	0.024
Adjuvant therapy (no vs. yes)	1.783	0.794-4.001	0.161
Ki67 (<10% vs. ≥10%)	2.683	1.552-4.639	<0.001
PHH3 (<5/10HPF vs. ≥5/10HPF)	0.949	0.556-1.621	0.848
IDH (wild-type vs. mutant)	0.368	0.244-0.556	<0.001
1p/19q Codeleted (no vs. yes)	0.275	0.131-0.575	0.001

### Validation of CHI3L2 mRNA Expression Levels and Prognostic Effect in TCGA and CGGA Datasets

**Figure 4:**
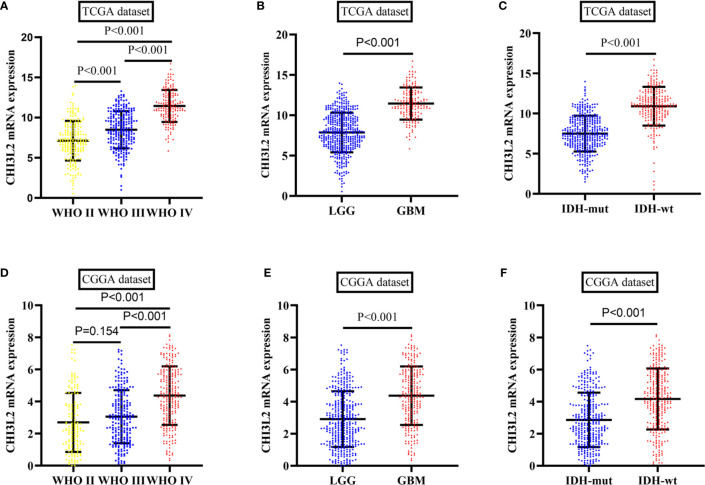
The mRNA expression levels of CHI3L2 in gliomas in TCGA and CGGA datasets. **(A)** CHI3L2 mRNA levels in diffusely infiltrating glioma (WHO II-IV) in the TCGA dataset. **(B)** CHI3L2 mRNA levels in LGG and GBM in TCGA dataset. **(C)** CHI3L2 mRNA levels in diffusely infiltrating glioma with different IDH status in TCGA dataset. **(D)** CHI3L2 mRNA levels in diffusely infiltrating glioma (WHO II-IV) in the CGGA dataset. **(E)** CHI3L2 mRNA levels in LGG and GBM in CGGA dataset. **(F)** CHI3L2 mRNA levels in diffusely infiltrating glioma with different IDH status in CGGA dataset.

**Figure 5:**
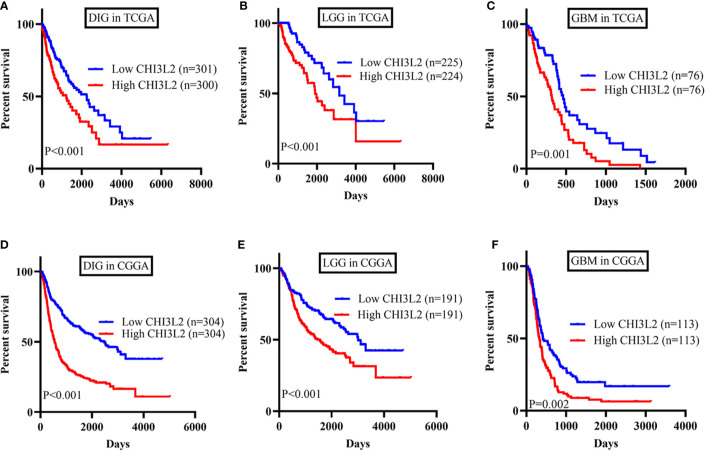
The prognostic impact of CHI3L2 mRNA levels in gliomas in TCGA and CGGA datasets. Kaplan-Meier curves reveal high CHI3L2 mRNA levels predict short overall survival time in diffusely infiltrating glioma (DIG), LGG, and GBM in the TCGA dataset **(A–C)** and CGGA dataset **(D–F)**.

To further verify the results of our study, we collected a total of 601 glioma samples from the TCGA dataset and 608 glioma samples from the CGGA dataset to analyze the CHI3L2 mRNA expression. In the TCGA dataset, CHI3L2 mRNA levels were significantly increased in GBM (WHO IV) compared with WHO II, WHO III, and LGG patients (**Figures 4A, B**). CHI3L2 mRNA expression levels were significantly higher in IDH wild-type gliomas compared with IDH mutant gliomas (**Figure 4C**). Similar results were also obtained in the CGGA dataset (**Figures 4D–F**). We further analyze the CHI3L2 mRNA expression levels in new molecular classification of diffusely infiltrating glioma, including IDH mutant without 1p/19q codeleted gliomas, IDH mutant with 1p/19q codeleted gliomas, and IDH wild-type gliomas, in TCGA and CGGA database (**Figure S4A** and **Figure S4B**). We found CHI3L2 mRNA expression levels in IDH wild-type gliomas are higher than IDH mutant gliomas. Gliomas with IDH mutant and 1p/19q codeleted have higher CHI3L2 mRNA levels than gliomas with IDH mutant and non-1p/19q codeleted. Moreover, we performed Kaplan-Meier analysis to confirm whether CHI3L2 mRNA levels could predict poor prognosis of gliomas in datasets. As shown in **Figure 5**, patients with high CHI3L2 mRNA levels correspond to shorter survival time in all glioma subgroups both in the TCGA (**Figures 5A–C**) and CGGA (**Figures 5D–F**) datasets. Similarly, we also verified the effect of CHI3L2 on the prognosis in the new molecular classification of glioma in database (**Figure S5**). Except for gliomas with IDH mutant and non-1p/19q codeleted in the TCGA dataset, high levels of CHI3L2 mRNA in any other subgroup indicate a poor prognosis, whether in the TCGA or CGGA dataset.

### Predicted Functions and Pathways of CHI3L2 in Gliomas

**Figure 6:**
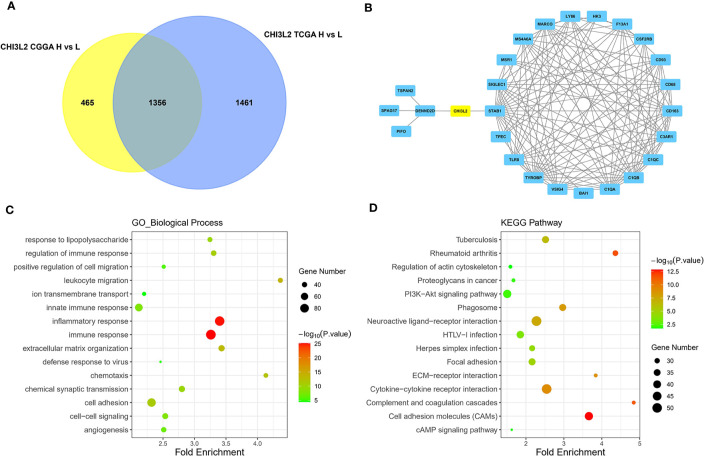
Identification of DEGs, hub genes, functions, and pathways of CHI3L2 in gliomas. **(A)** The Venn diagrams show a total of 1356 overlapping DEGs identified from TCGA and CGGA datasets. **(B)** The 26 hub genes are screened out by the cytoHubba plugin of Cytoscape software. **(C)** GO analysis shows multiple biological processes of the overlapping DEGs. **(D)** Several pathways of the overlapping DEGs are identified by KEGG analysis.

The GBM and LGG RNA-seq data were from TCGA and CGGA datasets. Limma package in R software was conducted to screen out the differentially expressed genes (DEGs) with the cut-off criterion of adjusted P< 0.05 and |log2FC| > 1. We identified 1356 overlapping DEGs which were aberrantly expressed in TCGA and CGGA datasets (**Figure 6A**). The top 26 hub genes were screened via the plug-in molecular complex detection and cytoHubba of Cytoscape (**Figure 6B**). GO analysis was performed to show the overlapping DEGs were involved in several biological processes, including angiogenesis, immune, and inflammatory response (**Figure 6C**). The KEGG pathways enriched in several classic signaling pathways, such as cell adhesion molecules (CAMs) and PI3K-Akt signaling pathways (**Figure 6D**).

### The Correlation Between CHI3L2 and Markers of Immune Infiltrates in Gliomas

**Figure 7:**
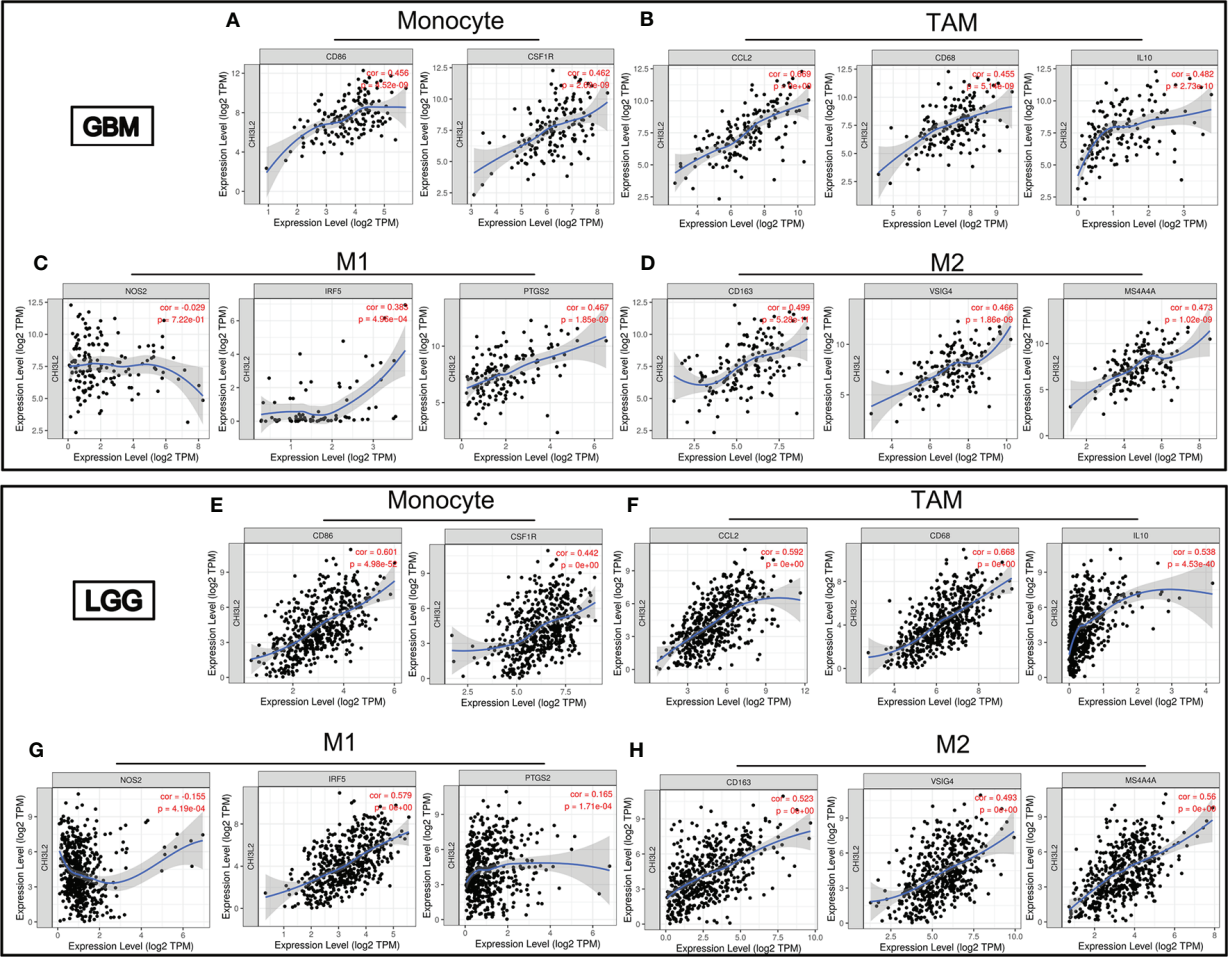
CHI3L2 expression correlates with marker sets of immune cells in GBM and LGG. Scatter plots illustrate correlations between CHI3L2 and markers of monocytes (CD 86 and CSF1R), TAMs (CCL2, CD68 and IL10), M1 (NOS2, IRF5, and PTGS2), and M2 macrophages (CD163, VSIG4, and MS4A4A) in GBM **(A–D)** and LGG **(E–H)**.

Infiltrating immune cells are important components of the tumor microenvironment and are frequently associated with tumor behavior and patient outcomes. Since GO analysis revealed that CHI3L2 was related to the immune response, we further explored the infiltration of immune cells in gliomas. To estimate the relevance of CHI3L2 and diverse immune cell markers, we used the TIMER platform to investigate correlations between CHI3L2 levels and markers of diverse immune cells, included monocytes, TAMs (tumor-associated macrophages), M1 and M2 macrophages, Tregs (regulatory T cells), exhausted T cells, CD8+ T cells, T cells (general), B cells and neutrophils in GBM and LGG (**Table 4**). We found CHI3L2 was significantly associated with marker sets of monocytes, TAMs, and M2 macrophages in GBM and LGG. Particularly, we showed the scatter plots of association between CHI3L2 and the marker sets of monocytes, TAMs, M1 phenotype, and M2 phenotype in GBM and LGG (**Figures 7A–H**). We also found significant correlations between CHI3L2 and some markers of Treg and T cell exhaustion, such as TGFβ1, CTLA4, TIM-3, and GZMB. Since Treg and T cell exhaustion play an important role in tumor immune escape. We believe CHI3L2 may also play an immunomodulation role in gliomas. In addition, we used several clinical commonly immune cell markers, including CD163, CD4, CD8, CD20, to perform immunohistochemical test on glioma samples, and found that CHI3L2+ macrophages have a certain correlation with CD163+ M2 macrophages (r=0.547, p<0.001), CD4+ T cells (r=0.330, p<0.001), CD8+ T cells (r=0.389, p<0.001), and CD20+ B cells (r=0.237, p<0.001) in gliomas (**Figure S6**).

**Table 4:** Correlation analysis between CHI3L2 and markers of immune cells in GBM and LGG.

Description	Gene markers	GBM		LGG	
		Cor	P	Cor	P
Monocyte	CD86	0.456	***	0.601	***
	CD115 (CSF1R)	0.462	***	0.442	***
TAM	CCL2	0.669	***	0.592	***
	CD68	0.455	***	0.668	***
	IL10	0.482	***	0.538	***
M1 Macrophage	INOS (NOS2)	-0.029	0.722	-0.155	***
	IRF5	0.383	***	0.579	***
	COX2 (PTGS2)	0.467	***	0.165	***
M2 Macrophage	CD163	0.499	***	0.523	***
	VSIG4	0.466	***	0.493	***
	MS4A4A	0.473	***	0.560	***
Treg	FOXP3	0.098	0.228	-0.084	0.056
	CCR8	0.151	0.062	0.183	***
	STAT5B	-0.030	0.714	-0.110	*
	TGFβ1	0.315	***	0.526	***
T cell exhaustion	PD-1 (PDCD1)	0.039	0.629	0.539	***
	CTLA4	0.323	**	0.381	***
	LAG3	-0.144	0.076	0.201	***
	TIM-3 (HAVCR2)	0.456	***	0.630	***
	GZMB	0.318	**	0.352	***
T cell (general)	CD3D	0.303	***	0.572	***
	CD3E	0.281	***	0.615	***
	CD2	0.319	***	0.626	***
CD8+ T cell	CD8A	-0.002	0.984	0.316	***
	CD8B	0.149	0.067	0.289	***
B cell	CD19	0.174	*	0.375	***
	CD79A	0.015	0.859	0.257	***
Neutrophils	CD66b (CEACAM8)	-0.101	0.214	0.014	0.756
	CD11b (ITGAM)	0.451	***	0.514	***
	CCR7	0.203	*	0.412	***

Cor, R-value of Spearman’s correlation. (*P < 0.05; **P < 0.01; ***P < 0.001).

### The Expression of CHI3L2 in Glioma Cell Lines and Its Effect on CD8+ T Cells

**Figure 8:**
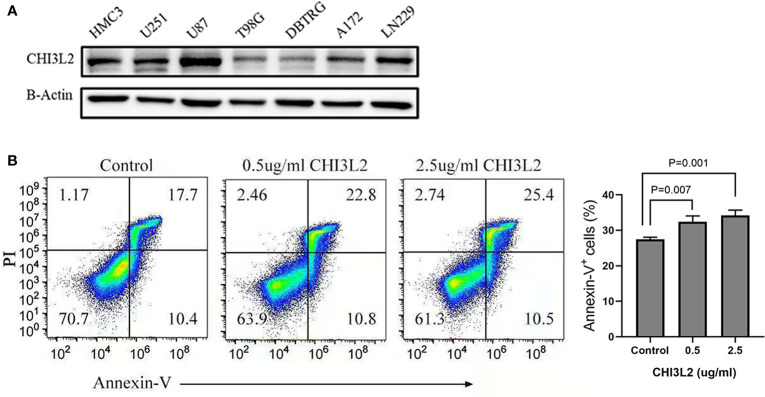
The expression of CHI3L2 was verified by Western Blot and the proportion of CD8+ T cell apoptosis was analyzed by flow cytometry. **(A)** Western blot of seven human glioma cell lines and one human microglia cell line (HMC3) reveals robust CHI3L2 protein expression. **(B)** CD8+ T cells were cultured with 10% serum media and treated with or without CHI3L2 for 72 hours. Cell apoptosis was analyzed by using FITC-annexin V-based flow cytometry, the cellular apoptotic rate was a sum of early and late apoptotic rates. Left, the representative sorting. Right, quantified result of three independent experiments.

The expression of CHI3L2 in glioblastoma cells (U251, U87, T98G, DBTRG, A172, LN229) and microglia cell (HMC3) has been verified by Western Blot (**Figure 8A**). **Figure 8A** shows that CHI3L2 is expressed in glioblastoma cell lines and a microglia cell line. It is strongly expressed in the glioblastoma cell lines U87, U251, LN229, A172 and the microglia cell line HMC3, while the expression in the glioblastoma cell lines T98G and DBTRG is weak. **Figure 8B** is the result of flow cytometry analysis. The left image is a representative sorting that lists the percentage of cells in each quadrant: bottom left-live cells; top left-mechanically damaged cells; bottom right-early apoptosis; top right- late apoptosis. The cellular apoptotic rate was a sum of early and late apoptotic rates. The proportion of apoptotic cells in the control group was 28.1%, the proportion of apoptotic cells in the 0.5ug/ml CHI3L2 group was 33.6%, and the proportion of apoptotic cells in the 2.5ug/ml CHI3L2 group was 35.9%. The right chart shows the percentage of apoptotic cells of three independent experiments. The result of flow cytometric analysis demonstrated that the apoptotic proportion of CD8+ T cells increases with increasing CHI3L2 concentration, indicating CHI3L2 may be related to immunosuppression.

## Discussion

At present, the outcome of most glioma is very poor, even with the use of comprehensive treatment strategies. It has been widely reported that the therapeutic resistance of glioma is closely related to its unique metabolic mechanism and the surrounding complex immunosuppressive microenvironment (32–34). Therefore, exploring reliable prognostic biomarkers and personalized treatment strategies for this disease are urgently needed. In the present study, CHI3L2 has been identified as a novel prognostic biomarker and associated with tumor immune infiltration markers in gliomas, which indicate CHI3L2 may serve as a target for glioma treatment in the future.

CHI3L2, as a member of the glycoside hydrolases 18 family, can act as a cytokine and growth factor but lacks chitinase activity (17). It was found produced by tumor-associated macrophages in breast cancer (13, 14). In renal cell carcinoma, CHI3L2 was mainly expressed in tumor cells and tumor-associated macrophages (15). Previous studies revealed CHI3L2 significantly increased in glioblastoma by northern blot hybridization and western blotting analysis and activated signal-regulated kinases ERK1/ERK2 leading to the initiation of MAP kinase signaling cascade in 293 and U87 MG cells (18, 26). However, the correlations between CHI3L2 expression and clinicopathological features, the association with tumor-infiltrating immune cells, the prognostic value of CHI3L2, and its other functions in gliomas are still unknown.

Our study showed CHI3L2 expressed in tumor cells and macrophage cells in glioma tissues and particularly up-regulated in GBM and IDH wild-type gliomas. The Kaplan-Meier curves reveal higher CHI3L2 expression levels correlated with short overall survival in diffusely infiltrating glioma, lower-grade glioma, and IDH wild-type gliomas. High CHI3L2 expression indicates a poor prognosis for glioma patients, regardless of whether the MGMT promoter is methylated or has received adjuvant therapy. Cox proportional hazards regression model indicates CHI3L2 expression in tumor cells is an independent prognostic indicator of glioma. TCGA and CGGA datasets further confirm our findings. However, it should be pointed out that no significant differences between high CHI3L2 and poor prognosis in GBM were achieved in our cohort, which is different from the results (The relationship between high CHI3L2 mRNA expression and short overall survival time were statistically significant in all subgroups) in TCGA and CGGA datasets. We believe there are two reasons for the inconsistent results. One probable reason is the difference in detection levels. The relative expression levels of CHI3L2 mRNA were detected using high-throughput sequencing in TCGA and CGGA datasets, but CHI3L2 expression levels in our samples were assessed at protein levels by immunohistochemistry. Another possible reason is the difference in sample size. Accordingly, we intend to enlarge our sample size in the following study.

Based on these results, we further performed GO and KEGG pathway analyses to conclude the CHI3L2-related genes were involved in several biological processes, including angiogenesis, immune, and inflammatory response, and enriched in several classic signaling pathways, including cell adhesion molecules and PI3K-Akt signaling pathway. It has been reported that CHI3L2 acts as a powerful monocyte chemotactic factor and angiogenesis stimulating factor in breast cancer (14). A recent review also reported CHI3L2 acts as a new target for anti-angiogenic therapy in breast cancer patients (35). The angiogenesis function of CHI3L2 may be responsible for the poor prognosis of glioma, which needs further confirmation by follow-up studies. A recent review reported the role of cell adhesion molecules (CAM) in immune responses and tumor microenvironment----Cell adhesion molecules affect the antigen-presenting function, and inhibit the development of regulated cells and the leaching of regulatory cells into tumors, thus promoting tumor immune escape (36). Our study showed CHI3L2 expressed in tumor cells and macrophages in glioma tissues. A previous study suggests human glioma- infiltrating macrophages have similar functions to CAM in mediated immune responses (37). It was also reported that CAMs are potential prognostic biomarkers and attractive therapeutic targets for glioblastoma (38). A previous study suggested PI3K-Akt signaling pathway activation, to some extent, affects the activity of most immune cell types. PI3K-AKT-mTOR pathway plays a certain role in regulating immunosuppression in tumor microenvironment (39). We speculate CHI3L2 may be able to act as immunomodulation through this pathway. Additionally, we have learned in previous studies that CHI3L1 (the homologous gene of CHI3L2) may be used as an immunomodulatory factor to affect the therapeutic efficacy of PI3K/AKT-based pathway inhibitors in glioblastoma (40). CHI3L1 also plays a key role in inducing immunosuppression and metastasis in breast cancer. CHI3L1 up-regulates pro-inflammatory mediators, CCL2, CXCL2 and MMP-9, all of which contribute to tumor growth and metastasis, and treatment with chitin can significantly reduce these effects (23). These studies provide a reference for us to further explore the internal mechanism of CHI3L2 in immune infiltration.

Based on the analysis of the TIMER platform, the correlations between CHI3L2 and markers of immune cells imply CHI3L2 may play a part in immunomodulation in GBM and LGG. Our results suggest CHI3L2 expression has strong correlations with marker sets, include CD86 and CSF1R of monocytes, CCL2, CD68, and IL 10 of TAMs and CD163, VSIG4, and MS4A4A of M2 macrophages in GBM and LGG. A study has shown that purified CHI3L2 strongly induces the migration of freshly isolated human CD14+ monocytes (14). It was reported that CD163 could act as a regulator of immune response and the potential to be a target to suppress immune escape and recover the function of T-cell populations in gliomas (41). In our experiments, we also found a strong correlation between CHI3L2 and CD163 (**Figure S6**). Additionally, there was a significant relationship between CHI3L2 and marker genes of Treg and T cell exhaustion, including TGFβ1, CTLA4, TIM-3, and GZMB. In the tumor microenvironment, TGFβ can serve as an anti-tumor immunosuppressive factor and play an essential part in Treg cells (42). It was reported that only TGFβ, the key regulatory factor of tumor progression, was able to stimulate CHI3L2 mRNA levels in human macrophages in vitro (43). TGF-β, which can be secreted by both microglial cells and glioma cells, participates in the functional transformation of macrophages into immunosuppressive and pro-invasive phenotypes, which supports tumor growth (44, 45). Macrophages are believed to be activated microglia within the central nervous system. Our data show that CHI3L2 is expressed in tumor cells and macrophages in glioma tissues, and has a certain correlation with TGF-β, further suggesting that CHI3L2 may also have the function of inhibiting tumor immune regulation and promoting tumor growth. Similarly, CTLA4 and TIM-3 can induce T cell exhaustion via direct mechanisms through the interactions with their ligands, leading to impaired T cell activation, inhibition of T cell proliferation, and impaired cytokine release (46). The correlation between CHI3L2 and T cell exhaustion markers indicates CHI3L2 may play a part in mediating T cell depletion. Our flow cytometry results further confirmed that CHI3L2 can induce CD8+ T cell apoptosis, indicating that CHI3L2 has the potential to promote tumor immune escape. However, how CHI3L2 protein promotes the apoptosis of CD8+ T cells needs to be further explored in future research.

The limitations of this study are as follows: First, the sample size of gliomas for IHC is limited. Second, we did not accurately define the type of CHI3L2 + macrophages. In addition, further experimental investigation and analysis are needed to gain insights into the underlying mechanisms.

In conclusion, our study suggests CHI3L2 may be a promising prognostic biomarker that contributes to poor prognosis for gliomas. CHI3L2 may also play an important role in immunomodulation, suggesting CHI3L2 may serve as a novel therapeutic target for glioma patients.

## Data Availability Statement

The raw data supporting the conclusions of this article will be made available by the authors, without undue reservation.

## Ethics Statement

The studies involving human participants were reviewed and approved by Sun Yat-Sen University Cancer Center. Written informed consent to participate in this study was provided by the participants' legal guardian/next of kin.

## Author Contributions

LL and WH designed the study and wrote the manuscript. LL, WH, JH, HD, and LS acquired the data. YY and HD provided help for the IHC test and Flow cytometry analysis. LL, YY, and WH performed the data analysis. WH and JZ reviewed the manuscript. All authors contributed to the article and approved the submitted version.

## Conflict of Interest

The authors declare that the research was conducted in the absence of any commercial or financial relationships that could be construed as a potential conflict of interest.

## Abbreviations

CHI3L2, Chitinase-3-Like Protein 2; CGGA, Chinese Glioma Genome Atlas; CLPs, chitinase-like proteins; Cor, R-value of Spearman's correlation; CAMs, cell adhesion molecules; DEGs, differentially expressed genes; DIG, diffusely infiltrating glioma; FISH, fluorescent in situ hybridization; GBM, glioblastoma multiforme; GO, Gene Ontology; IHC, immunohistochemistry; IDH, isocitrate dehydrogenase; KEGG, Kyoto Encyclopedia of Gene and Genomes; LGG, lower-grade glioma; TAM, tumor-associated macrophage; TCGA, The Cancer Genome Atlas; TIMER, Tumor IMmune Estimation Resource; Treg, regulatory T cell; WHO, World Health Organization.
