# Supplementary material for: CHI3L2 Is a Novel Prognostic Biomarker and Correlated With Immune Infiltrates in Gliomas
Source: Front Oncol. 2021 Apr 15;11:611038. doi: 10.3389/fonc.2021.611038 (PMC8084183; doi:10.3389/fonc.2021.611038)
Supplement: Supplementary file 1 [file DataSheet_1.docx]

Supplementary Material

**1 Supplementary Tables and Figures**

## 1.1 Supplementary Tables

**Table S1.** The clinical characteristics of all 288 glioma patients.

| Characteristic | N=288 |
| --- | --- |
| Grade |  |
| WHO II | 81 |
| WHO III | 76 |
| WHO IV | 131 |
| Histology |  |
| Astrocytoma | 112 |
| Oligodendroglioma | 45 |
| Glioblastoma | 131 |
| Gender |  |
| Male | 167 |
| Female | 121 |
| Age (years) |  |
| Median | 43 |
| Range | 7-78 |
| Location |  |
| Supratentorial | 278 |
| Infratentorial | 10 |
| Adjuvant therapy |  |
| Yes | 264 |
| No | 24 |
| Overall survival (months) |  |
| Median | 27 |
| Range | 0-110 |

**1.2 Supplementary Figures**


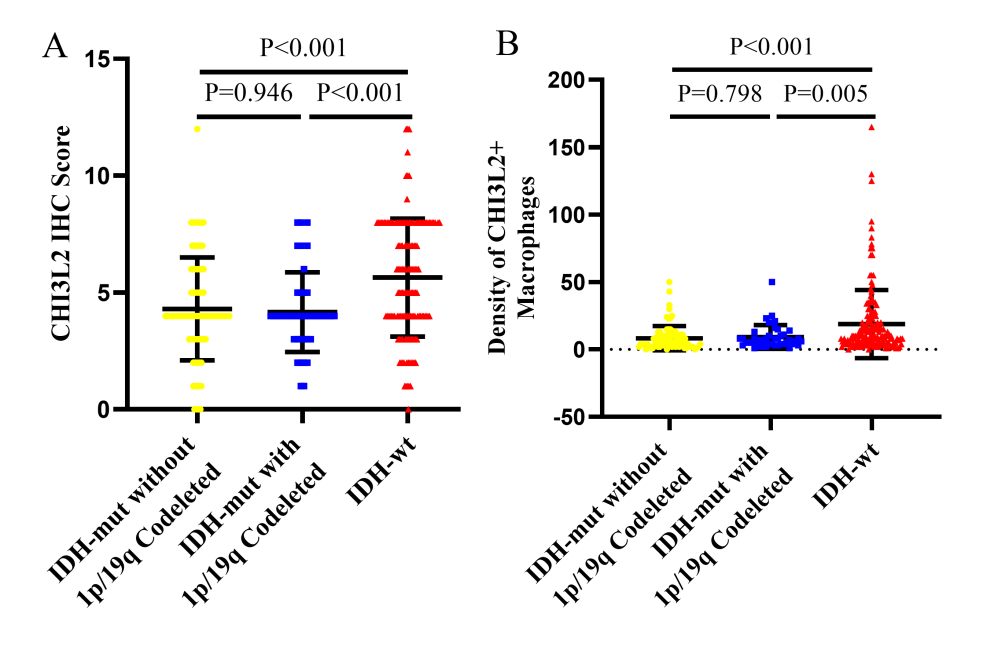


**Figure S1.** The protein expression levels of CHI3L2 in different subgroups of gliomas. The CHI3L2 IHC score **(A)** and density of CHI3L2+ macrophages **(B)** in diffusely infiltrating glioma with different IDH mutation and 1p/19q codeletion status.


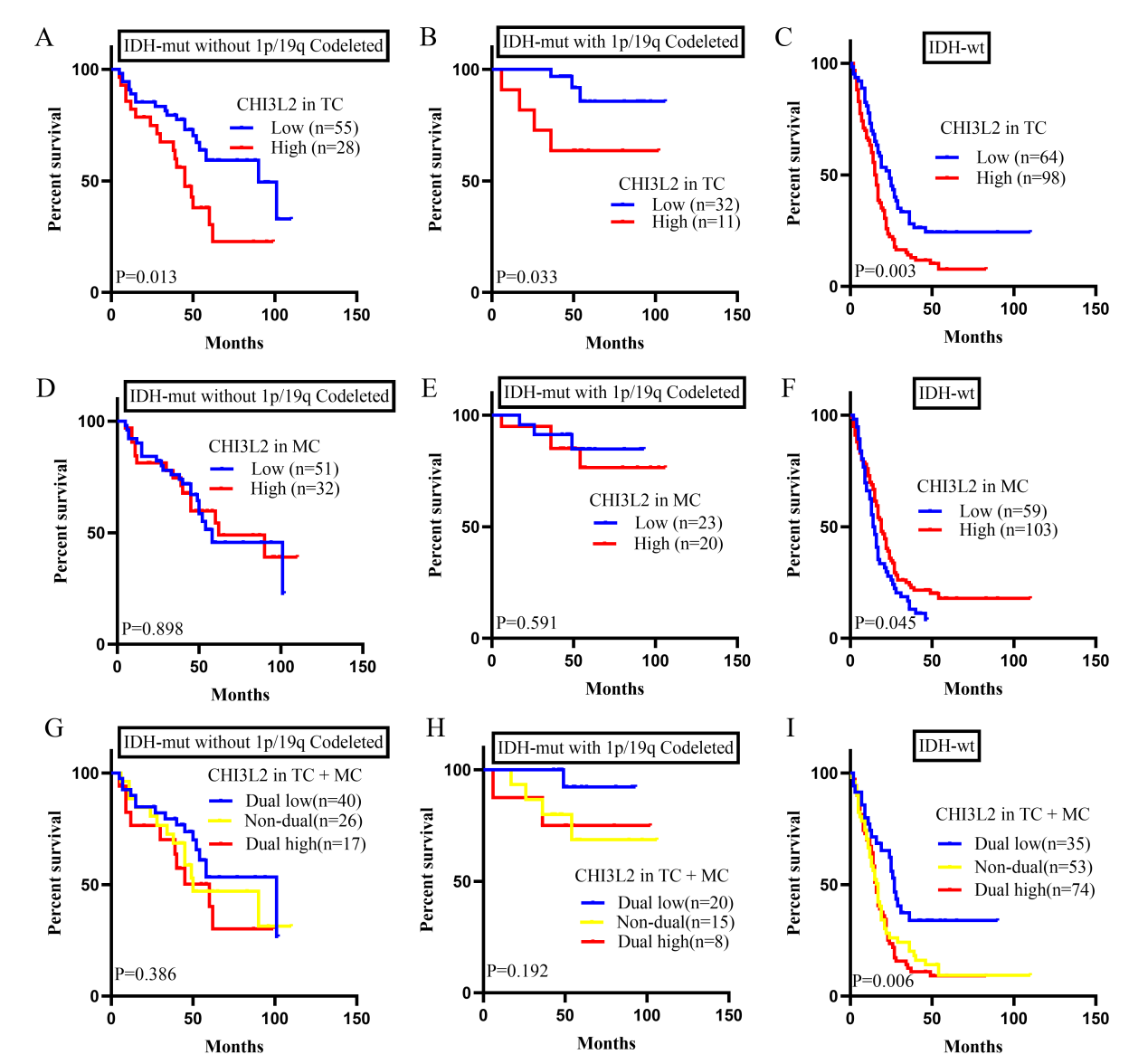


**Figure S2.** CHI3L2 protein expression affects overall survival (OS) in glioma patients. Kaplan-Meier curves showing a correction of CHI3L2 expression with OS in tumor cells **(A-C)**, macrophage cells **(D-F)**, and tumor cells + macrophages cells **(G-I)** in glioma of IDH-mutant without 1p/19q codeleted, IDH-mutant with 1p/19q codeleted, and IDH wild type.


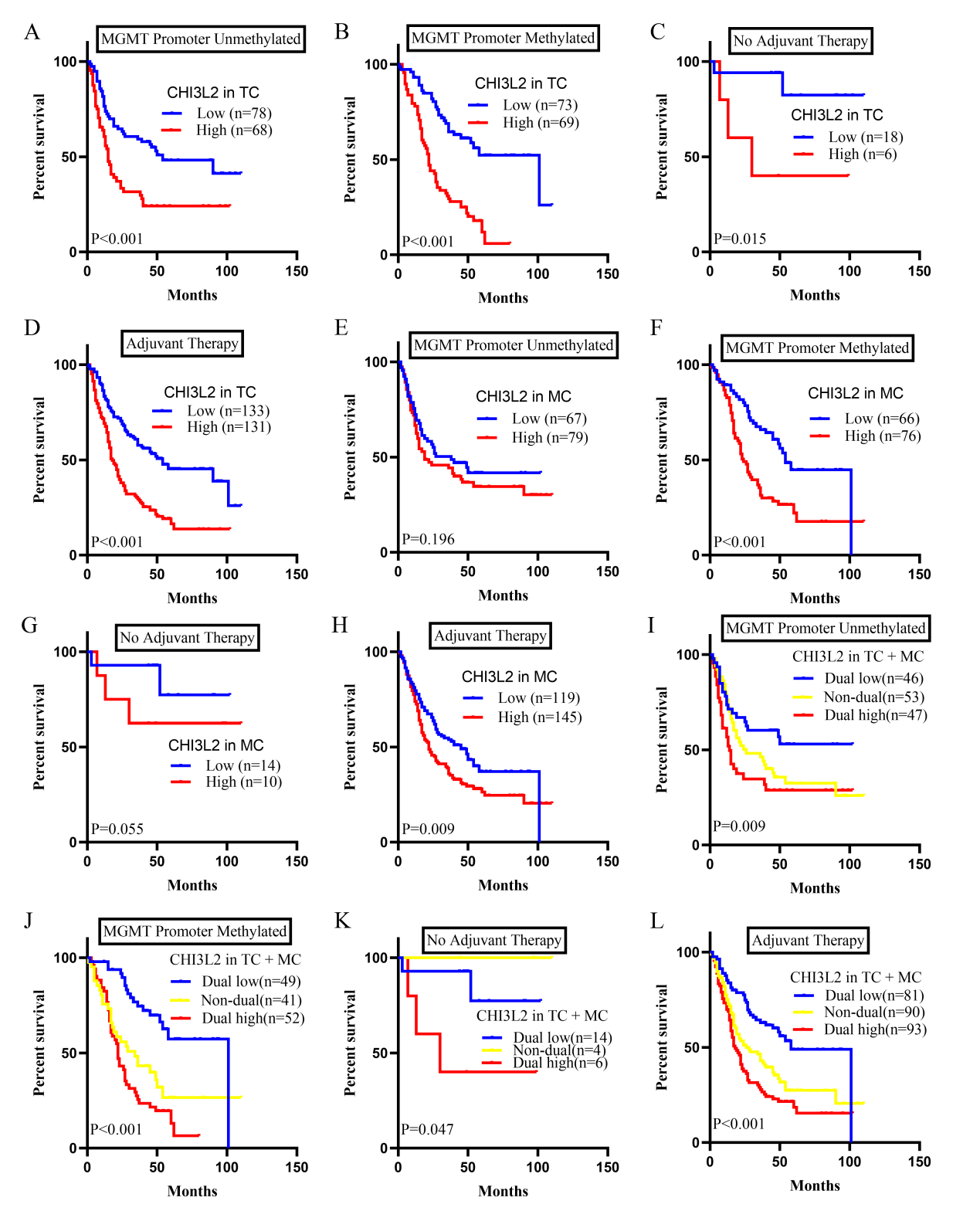


**Figure S3.** The prognostic effect of CHI3L2 in patients with different MGMT promoter methylation and adjuvant therapy status. Kaplan-Meier curves showing a correction of CHI3L2 expression with OS in tumor cells **(A-D)**, macrophage cells **(E-H)**, and tumor cells + macrophages cells **(I-L)** in glioma with MGMT promoter unmethylated, MGMT promoter methylated, no adjuvant therapy, and adjuvant therapy.


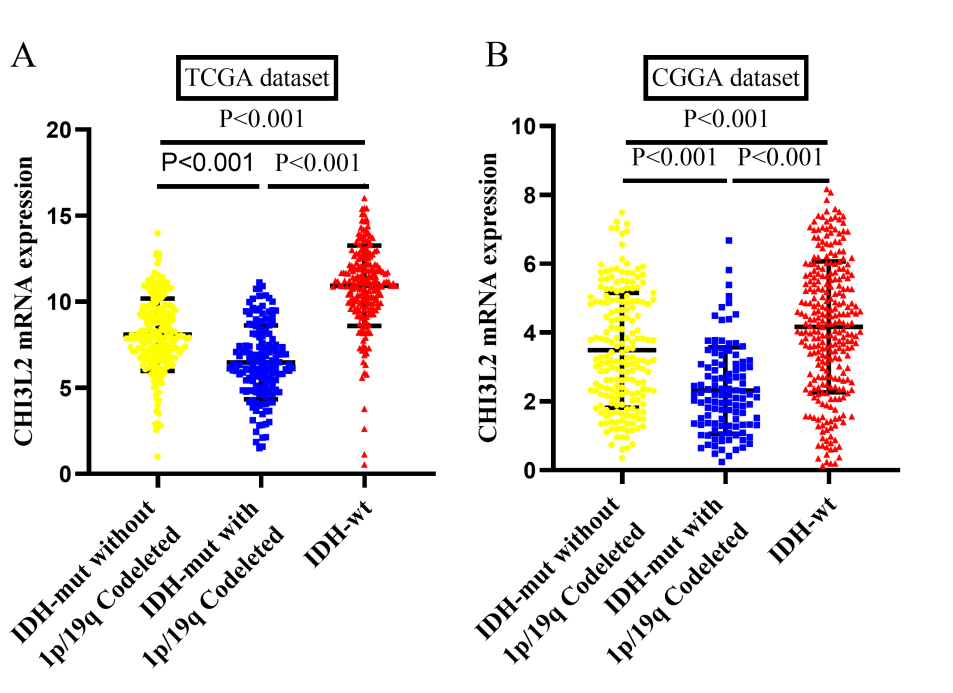


**Figure S4.** The mRNAexpression levels of CHI3L2 in diffusely infiltrating glioma with different IDH mutation and 1p/19q codeletion status in TCGA **(A)** and CGGA **(B)** datasets.


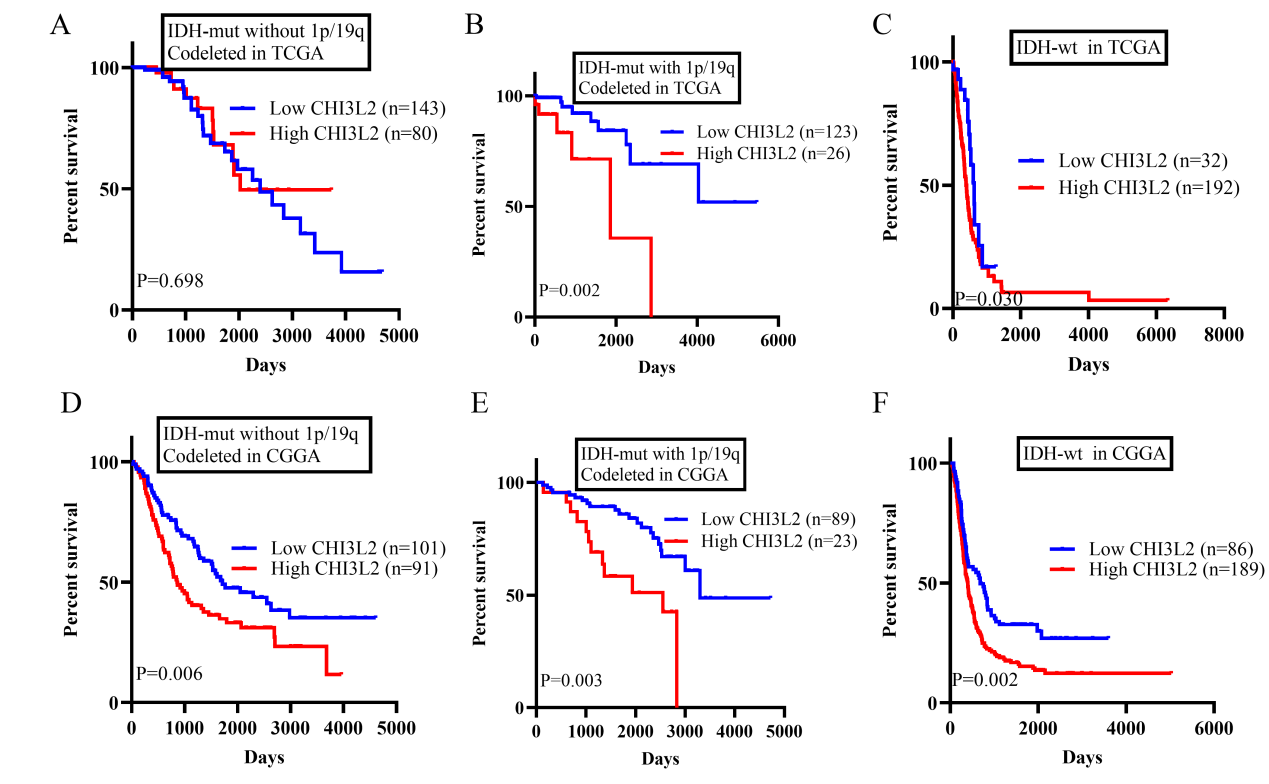


**Figure S5.** CHI3L2 mRNA levels affect overall survival (OS) in glioma patients. Kaplan-Meier curves showing a correction of CHI3L2 mRNA levels with OS in gliomas of IDH-mutant without 1p/19q codeleted, IDH-mutant with 1p/19q codeleted , and IDH wild type in TCGA **(A-C)** and CGGA **(D-F)** datasets.


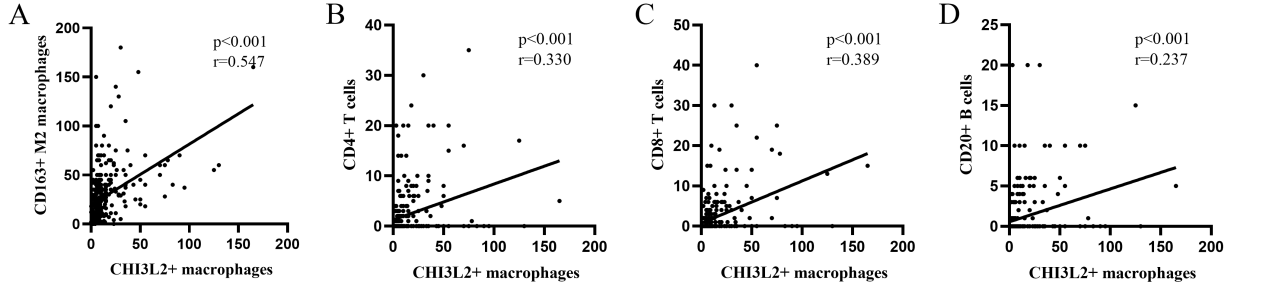


**Figure S6.** Correlations between the density of CHI3L2+ macrophages and CD163+ M2 macrophages **(A)**, CD4+ T cells **(B)**, CD8+ T cells **(C)**, and CD20+ B cells **(D)** in gliomas were calculated with Spearman's rank correlation coefficient.
